# Exploring Oxidative Stress in Different Endometriosis Phenoptypes: Insights from Ovarian and Systemic Perspectives by the Study of SIRT3

**DOI:** 10.3390/ijms26189110

**Published:** 2025-09-18

**Authors:** Anna Goday, Laura Valls-Roca, Marta Méndez, Yolanda Cívico, Meritxell Gràcia, Mariona Guitart-Mampel, Gemma Casals, Sara Peralta, Aina Borrás, Francisco Fàbregues, Inés Agustí, Yasmina Barral, Cristina Ros, Maria Àngels Martínez, Mariona Rius, Salvadora Cívico, Gloria Garrabou, Francisco Carmona, Dolors Manau

**Affiliations:** 1 Assisted Human Reproduction Unit, Gynecology Service, Clinic Institute of Gynecology, Obstetrics and Neonatology (ICGON), Hospital Clínic, 08036 Barcelona, Spain; anngod@dexeus.com (A.G.); aborras1@clinic.cat (A.B.); fgasol@clinic.cat (F.F.);; 2 Faculty of Medicine and Health Sciences, Universitat de Barcelona (UB), 08036 Barcelona, Spaingarrabou@clinic.cat (G.G.);; 3 Inherited Metabolic Diseases and Muscular Disorders Research Group, Cellex-IDIBAPS, Faculty of Medicine and Health Sciences-UB, Hospital Clinic of Barcelona, 08036 Barcelona, Spain; 4 CIBERER—Spanish Biomedical Research Centre in Rare Diseases, 28029 Madrid, Spain; 5 Institut d’Investigacions Biomèdiques August Pi i Sunyer (IDIBAPS), C/Villarroel 170, 08036 Barcelona, Spain; 6 Gynecology Department, Institute Clinic of Gynecology, Obstetrics and Neonatology (ICGON), Hospital Clinic of Barcelona, Universitat de Barcelona, 08036 Barcelona, Spain

**Keywords:** endometriosis, oxidative stress, sirtuins, SIRT3, infertility, in vitro fertilization (IVF), deep endometriosis, ovarian endometrioma

## Abstract

Endometriosis affects about 10% of reproductive-aged women, characterized by endometrial-like tissue outside the uterus, leading to chronic inflammation. The exact cause remains unknown, though genetic and epigenetic factors are increasingly recognized alongside traditional theories. The disease manifests in forms such as endometriomas, whether superficial or peritoneal, and deep infiltrating lesions, often causing chronic pain and infertility. Infertility affects nearly 50% of patients, requiring expensive treatments like in vitro fertilization. Oxidative stress plays a key role in endometriosis, with sirtuins, especially SIRT3, emerging as important regulators. SIRT3, located in mitochondria, helps manage oxidative stress and redox balance. Despite extensive research, no diagnostic biomarkers exist. This longitudinal study compares oxidative stress markers and SIRT3 levels in patients with different endometriosis types. While classic oxidative stress markers showed no significant differences, higher SIRT3 levels were observed in peripheral blood mononuclear cells of patients with deep endometriosis. Additionally, patients with prior surgeries had elevated SIRT3 levels, indicating a possible link between disease severity and SIRT3 expression. The findings suggest SIRT3 as a potential therapeutic target in endometriosis management.

## 1. Introduction

Endometriosis is a chronic benign condition with a prevalence of around 10% in women of reproductive age and is characterized by the presence of endometrial-like tissue outside the uterine cavity, inducing a chronic inflammatory environment [1].

Endometriosis can present different phenotypes of lesions depending on the location and depth of the tissue involved such as ovarian endometrioma (OMA), peritoneal surface endometriosis or deep infiltrating endometriosis (DE) and often causes chronic pain which has a significant impact on the quality of life of patients, affecting daily activities, sexual life and mental health, and work productivity, leading to a high rate of absenteeism [2]. Additionally, up to 50% women with endometriosis experience fertility problems, with many requiring costly assisted reproduction techniques (ART), such as in vitro fertilization (IVF) to achieve pregnancy, underscoring the substantial economic impact of the disease. The mechanisms underlying endometriosis-related infertility are not fully understood but may involve factors such as anatomical distortion of the fallopian tubes, an altered follicular microenvironment [3,4,5], and dysregulation of steroid hormone metabolism and endometrial receptivity.

Numerous studies have focused on the role of oxidative stress in the progression of endometriosis, indicating an imbalance between free radicals and antioxidant molecules [6]. Different oxidative stress markers have been identified in the peritoneal fluid of women with deep endometriosis, in the follicular fluid of those with ovarian endometriomas, and in various other endometriosis-related samples. Furthermore, emerging evidence suggests that the oxidative stress profile may vary between these phenotypes, with deep endometriosis and endometriomas potentially exhibiting distinct oxidative stress responses due to differences in the local tissue environment and the extent of the disease [7].

In recent years, the study of novel stress regulators, which respond to redox alterations in reproductive cells, has gained traction. Among these, sirtuins (SIRT), a family of NAD+-dependent deacetylases, have emerged as crucial metabolic sensors of oxidative stress. Various subtypes of sirtuins, particularly SIRT1, SIRT3 and SIRT5, have been closely linked to female reproductive function, playing critical roles in folliculogenesis, steroidogenesis and embryo development. These molecules are expressed in mural granulosa cells (GCs) and cumulus cells (CC) of the Graafian follicle [8]. The exploration of these particles has significantly advanced our understanding of oxidative stress regulation in endometriosis, particularly at the ovarian level [9]. Taking into account that sirtuins act as sensors of cellular redox status, and considering that endometriosis is associated with oxidative stress and redox imbalance, the involvement of sirtuins in the pathophysiology of endometriosis is further supported. Many studies have related sirtuins with endometriosis, particularly Sirtuin 1 (SIRT1), which it has been suggested that may serve as potential biomarkers for endometriosis [10] or for example, in animal models it has been described as part of the pathogenesis [11]. Moreover, as Tatone et al. [8] explains, an upregulation of SIRT1 could contribute to a reduction in the expression of inflammatory cytokines.

Specifically, SIRT3 is primarily localized within the mitochondria and plays a pivotal role in mitochondrial metabolism. Its function in mitigating the accumulation of reactive oxygen species (ROS) has been extensively studied, implicating SIRT3 in various age-related pathologies. Notably, the enzymatic activity of SIRT3 promotes the deacetylation and activation of antioxidant enzymes, thereby maintaining redox homeostasis and preserving mitochondrial integrity. Beyond aging, dysregulation of SIRT3 has been linked to cardiovascular diseases, metabolic disorders, and cancer, underscoring its importance in the pathogenesis of multiple diseases [12]. Moreover, Sirtuin 3 (SIRT3) regulates mitochondrial antioxidant defenses through the activation of superoxide dismutase 2 (SOD2). This regulation occurs via deacetylation and activation of the transcription factor FOXO3a, which subsequently promotes the transcription of SOD2 [13]. Therefore, assessing the expression or activity levels of FOXO3a and SOD2 can serve as an indirect approach to evaluate SIRT3 activity.

Previous studies have explored the production of ROS by endometriotic lesions [14]; however, the aim of this study was to compare the oxidative stress profile at both local and systemic levels between ovarian and deep endometriosis in infertile women undergoing an IVF cycle. We sought to determine whether there is a local or systemic imbalance in ROS levels, focusing not only on classical ROS and antioxidant molecules but also on new oxidative stress regulators, both in ovarian follicular fluid and in blood. To our knowledge, this is the first preliminary study to compare the oxidative stress profiles between ovarian and deep endometriosis during an IVF cycle. This study aims to enhance the current understanding of the pathophysiology of endometriosis, providing insights that could lead to more targeted management strategies for this complex condition in the future.

## 2. Results

### 2.1. Patient Characteristics

After diagnosis by in depth examination with 3D TVUS, 20 patients with ovarian OMA alone, 20 patients with DE and without OMA, and 20 patients with both phenotypes were included in the study.

There were no statistically significant differences regarding epidemiological patient characteristics, such as age or BMI. However, patients with DE and MIX had a mean AFC significantly lower than OMA group (*p*-value: 0.01). No differences were found regarding the AMH (Table 1).

### 2.2. Pre-IVF Cycle Treatments

Although the difference did not reach a statistical significance, a greater consumption of antioxidants was observed in patients with the presence of DE (3 DE, 1 OMA and 8 MIX) (*p* = 0.128). On the other hand, these patients (DE and MIX) more frequently had previous surgery: 55% of DE, 11% of OMA and 100% of MIX (*p* < 0.001) and they also had more hormonal treatment: 84% in DE, 32% in OMA and 60% of MIX (*p* < 0.001) (Table 2). In most patients who had taken previous hormonal treatment, a wash out period was not performed before starting the ovarian stimulation (Table 2).

### 2.3. Outcomes of IVF Cycles

Regarding the results of the IVF cycle, patients with DE and MIX presented lower estradiol levels at the end of stimulation, although these levels did not reach statistical significance, and a lower number of mature oocytes compared to patients without pelvic endometriosis (*p* = 0.03). Despite this, the fertilization rate, the mean number of embryos transferred, and the useful embryo rate (defined as a good quality embryo per transfer or cryopreservation) were the same. Finally, although lower in patients with DE, the pregnancy rate was not significantly different (Table 3).

### 2.4. Results of Oxido-Reduction Markers

The study of the oxidation-reduction markers both at the systemic and ovarian level, and both in cellular (COCs or PBMCs) or liquid samples (FF/blood), showed no differences among the three groups (Table 4).

### 2.5. Oxidative Stress Regulator Results

**Figure 1:**
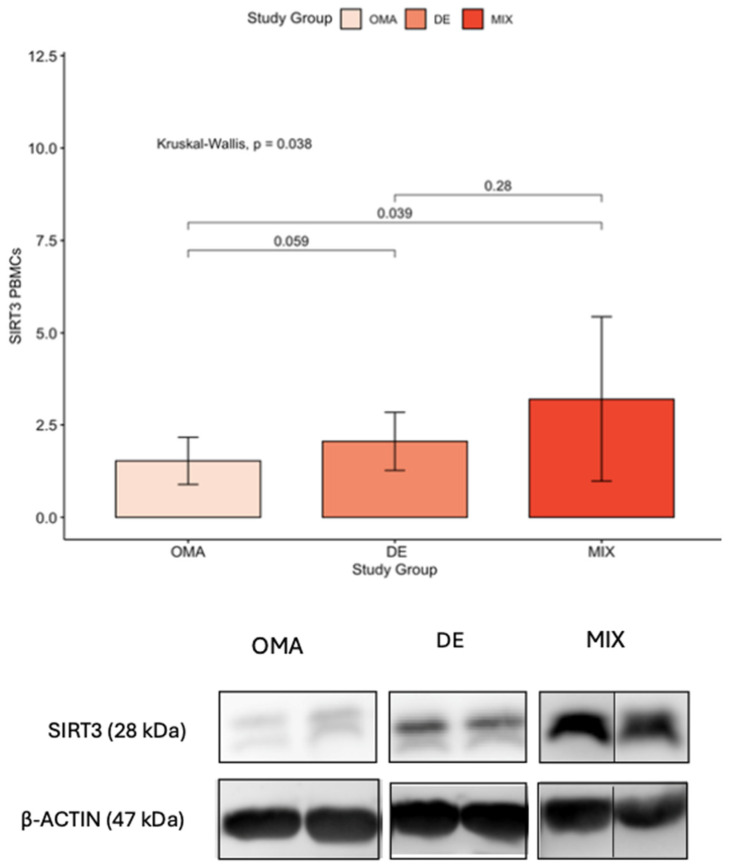
Expression levels of SIRT3 in PBMCs across the three study groups (OMA, DE, and MIX). Columns represent mean values, and whiskers indicate the standard deviation (SD). Statistical significance was assessed using the Kruskal–Wallis test followed by pairwise post hoc comparisons with Bonferroni correction. A *p*-value < 0.05 was considered statistically significant. The figure below includes a representative immunoblot showing SIRT3 expression for each group (OMA, DE, and MIX), alongside the corresponding β-actin loading control to confirm equal protein loading. The molecular weights of the detected proteins are indicated.

The oxidative stress regulators of the molecular pathways involved in the production of free radicals and antioxidants, showed similar results between the two groups, except for SIRT3 levels in the blood leukocyte population, which were statistically higher in patients with deep endometriosis (Figure 1, Table S1 of Supplementary Files).

Considering the elevation of SIRT3 levels in PBMCs observed in the most severe forms of endometriosis, we conducted a sub-analysis using logistic regression to assess whether this elevation was dependent on parameters such as prior antioxidant intake, pre-IVF hormonal therapy, or a history of previous endometriosis surgery. The results demonstrated that patients with a history of prior surgery for endometriosis exhibited significantly higher SIRT3 levels in PBMCs, a trend not observed in relation to antioxidant supplementation or prior hormonal therapy (Table 5).

**Figure 2:**
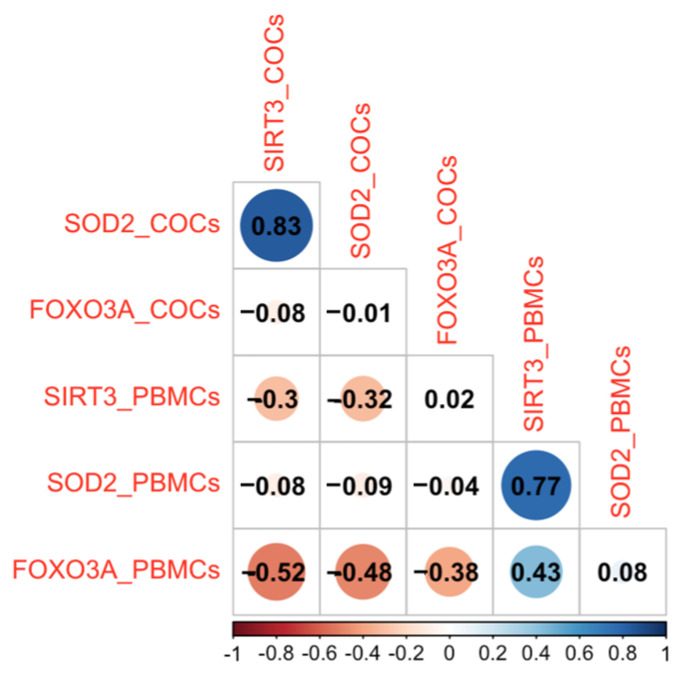
Correlation plot for the associations between molecular studied variables. The symmetric correlation matrix was created using the *R* “corrplot” package. The colors represent the degree of pairwise correlation according Pearson’s correlation coefficient.

We also performed an analysis of the most important correlations between different parameters, and our study revealed two positive correlations with particular significance: when SIRT3 levels were elevated, concurrent increases were observed in the expressions of SOD2 (*p*-value < 0.001, *R* = 0.83) at the COC level and the same correlation pattern is observed between SIRT3 and SOD2 at the PBMCs level (*p*-value = 0.018, *R* = 0.77). This finding suggests a potential regulatory relationship between SIRT3, SOD2, and FOXO3a, indicating their coordinated roles within the biological system (Figure 2).

## 3. Discussion

Endometriosis is a chronic disease with a complex pathophysiology, much of which remains to be explained today [15]. However, the pathogenesis of endometriosis is believed to be influenced by factors, such as chronic inflammation, hormonal imbalance [16], immunological dysfunction [17], and oxidative stress [18]. Regarding the latter factor, oxidative stress in endometriosis has been widely studied in recent decades. However, to our knowledge, this is the first study that compares the oxidative stress profile of infertile endometriosis patients at both a systemic and ovarian level, and in fluid and cellular samples.

In addition, considering that previous studies were based on classic stress markers, the main strength of the present study is that the levels of a novel regulator (SIRT3) were evaluated in infertile patients with endometriosis who underwent an IVF cycle. The presence of this oxido-reduction regulator was evaluated close to the target cell of this study, the oocyte, and also far from it at a systemic level. In this regard, this study was designed to assess possible differences in the oxidative stress profiles among patients with different endometriosis phenotypes: patients with OMA, and then patients with a more severe disease (both DE group without endometriosis in the ovary, and the MIX group, who had disease in the ovary and far from it).

The patients recruited for the study were comparable both in terms of their mean age and the absence of concomitant chronic diseases, such as diabetes or immunological diseases. The patients diagnosed with DE exhibited a different profile: poorer ovarian reserve markers (especially those with MIX endometriosis), had undergone significantly more surgical interventions due to a more symptomatic disease, were also significantly more frequently treated with hormonal treatments and showed a trend towards taking more antioxidant treatments. Hirokawa et al. showed that surgical interventions for severe endometriosis, albeit not of the ovary, could also affect the ovarian reserve due to the interference with the vascular network of the ovary [19]. Moreover, it is important to remark that the study was performed in a tertiary level hospital, with a recognized, expert multidisciplinary endometriosis unit, which consistently attends a substantial number of patients presenting severe endometriosis, persistent pain and infertility. According to the latest European Society of Human reproduction and Embryology guidelines on endometriosis, prior to an IVF cycle, women should be offered surgical excision of DE lesions mainly according to pain symptoms, as it is a good option to reduce endometriosis-associated pain [20]. This explains why the DE patients in the present study had undergone significantly more surgical interventions.

Most of the studies that talk about oxidative stress and endometriosis have recorded the presence of classical oxidative stress markers in the peritoneal fluid, in FF and in serum [21,22,23,24,25]. A study by Singh et al. compared the levels of classical oxidative stress markers, such as TAC, MDA or nitric oxide, among others, in FF in patients with advanced stages of endometriosis (grade III-IV ASRM classification) and patients with tubal sterility. They found that some markers, such as ROS, MDA and NO, were significantly higher in FF of women with advances stages of endometriosis [21]. By contrast, other authors, such as Prieto et al., reported significantly higher levels of vitamin E in plasma in women with endometriosis compared to women with other sterility causes, but were unable to explain these differences [24]. These studies demonstrate the contradictory results reported in the literature. Furthermore, most publications compare many oxidative stress markers between infertile patients with endometriosis and patients with other causes of sterility. Saito et al. studied 8-hydroxy-2′-deoxyguanosine (8-OHdG) in GCs in patients with endometriosis and in control patients without endometriosis and found higher levels of oxidative stress in the endometriosis patients [26]. The same occurred when Polak et al. studied 8-OHdG levels in peritoneal fluid in women with endometriosis and compared to women without endometriosis [23].

As shown above, the relationship between endometriosis and oxidative stress has been studied using different methodologies, patient heterogeneity, comparisons with populations without endometriosis, and classifications of endometriosis different from that used in our study and with many controversial results. For that reason, in order to try to homogenize the study groups, the present study compared the levels of oxidative stress markers among different endometriosis phenotypes.

This study found no significant differences in classical oxidative stress markers among the different types of endometriosis. Since it is known that DE is generally a more severe disease, we expected to find worse outcomes in the oxido-reduction markers. However, this was not the case. It should be noted that in the present study compared different groups of endometriosis without a control group. Additionally, the absence of differences among groups is likely due to the fact that these women had undergone more previous treatment, both hormonal and surgical, which could contribute to the unexpected outcomes and non-statistical differences observed in our analysis. Biasioli et al. [27] described more oxidative stress at a systemic level in patients with DE compared to those with OMA, but concluded that the DE patients who have received previous hormonal treatment benefit more in terms of oxidative stress markers [27]. On the other hand, in relation to metabolomics, recent studies have delineated a distinct profile in the FF of individuals with more severe endometriosis [5]. Nonetheless, our results differed in regard to oxido-reduction markers.

In reference to the sirtuins, they are NAD +- deacetylases and as Tatone et al. [8], stated: “*they have recently emerged as key metabolic sensors for body homeostasis*” [8]. It has been proven that sirtuins respond to metabolic changes, inflammatory signals, and oxidative stress, and are also associated with aging and longevity. Fertility is a process that is highly sensitive to an imbalance in oxidative stress associated with age and metabolic dysfunction. Consequently, reproductive cells undergo constant stress challenges and need adaptive responses, particularly when manipulated by ART. Hence, sirtuins have emerged as indispensable regulators of key processes in both oogenesis and spermatogenesis [8]. Within the sirtuin family, SIRT1, SIRT3 and SIRT5 are well documented as being expressed in the GCs and the CCs in humans and they are known to play an essential role in these reproductive cells [28,29].

Our study focused on SIRT3 because of its different location at the cellular level and because of its different roles in ovarian physiology. The predominant mitochondrial localization of SIRT3 makes it particularly relevant for investigating mitochondrial function and oxidative stress response, both of which are crucial aspects in the pathophysiology of endometriosis. SIRT3 has been implicated in maintaining mitochondrial homeostasis and enhancing antioxidant defenses. The function of SIRT3 is to activate SOD2 which helps to improve the efficacy of ROS removal [30].

The most important finding of our study was the significant elevation of SIRT3 levels in PBMCs in patients with DE compared to those with OMA (referring to endometriosis phenotype limited to the ovary). This observation, limited to the PBMCs, suggests a potential association between SIRT3 and the severity of endometriosis, particularly in cases in which the disease manifests as a combination of deep infiltrating and ovarian involvement.

The identification of elevated SIRT3 levels in PBMCs in DE, raises intriguing questions about the nature of this gynecological condition. SIRT3 is known for its role in mitochondrial function and cellular stress response, such as in Policystic Ovary Syndrome (PCOs) where it enhances insulin sensitivity and antioxidant defenses [8]. But it may also play a role in the pathophysiology of endometriosis, especially in cases in which the disease has deeply infiltrated surrounding tissues. The increased expression of SIRT3 in PBMCs of DE patients may suggest that this form of endometriosis may have a more systemic impact than previously thought which aligns with the notion that DE is the most severe form of endometriosis leading to more pronounced symptoms.

In line with our results, the study of Kokot et al., which compared sirtuins in serum samples between women with endometriosis and without endometriosis, found higher SIRT3 levels in women with advanced stages of endometriosis although these did not reach statistical significance [31]. Nonetheless, this study did not consider the day of the menstrual cycle in which the values were measured, nor was the study carried out in an infertile population. In addition, the samples were not cellular samples. In the same line of research, González-Fernández et al. observed intermediate levels of SIRT3 expression in GCs in infertile patients with advanced stages of endometriosis compared to infertile women with no ovarian factor, but again, these levels did not reach statistical significance, and SIRT3 was not evaluated outside of the ovary [32].

Our findings suggest that SIRT3 may be associated with the severity of endometriosis. However, this hypothesis requires further investigation, particularly through studies that include healthy control groups.

The finding that SIRT3 levels are elevated in patients with a history of previous endometriosis surgery raises interesting questions about the long-term impact of surgical interventions on oxidative stress. It could be interpreted as a compensatory mechanism to counteract the increased oxidative stress induced by the surgical procedure. Alternatively, it may reflect an underlying systemic response that persists long after the surgery, particularly in cases where the disease is recurrent or particularly aggressive. Moreover, the correlation observed between elevated SIRT3 and SOD2 levels further supports the idea of a coordinated antioxidant response in severe endometriosis, potentially mitigating oxidative stress despite the disease’s severity [8].

In addition, elevated SIRT3 levels in the blood cells of patients with deep endometriosis may reflect important alterations in cellular metabolism and mitochondrial homeostasis. By enhancing antioxidant defenses and preserving mitochondrial integrity, SIRT3 may contribute to the survival of ectopic endometrial cells in a hostile microenvironment. Furthermore, dysregulated SIRT3 activity would facilitate the invasive and fibrotic features of deep endometriosis [33,34]. These observations suggest that elevated SIRT3 play a functional role in the development and progression of the disease.

SIRT3 could also be an interesting therapeutic target in reproductive medicine. Studies have shown that higher levels of SIRT3 in human and mouse oocytes improve embryo development by reducing oxidative damage and preventing growth arrest [35]. In obese mouse models, activating SIRT3 improved oocyte quality by reducing ROS levels and restoring normal spindle formation. Other research suggests that SIRT3 helps slow down ovarian aging by protecting mitochondria and maintaining genomic stability [36]. Together, these findings indicate that SIRT3 may help reduce the negative effects of oxidative stress in endometriosis and could be a new approach to improve oocyte quality and IVF outcomes.

The absence of a control group without endometriosis and the small sample size are the greatest limitations of this study, warranting caution in generalizing the findings. However, previous studies based on the same line of study had similar sample sizes [9,27,31]. Despite its limitations, the present study represents a pioneering effort in comparing oxidative stress markers and regulators across different endometriosis lesions in infertile patients. This investigation extends beyond previous research by examining both systemic and ovarian levels of these markers in liquid and cellular samples.

A notable outcome of our study was the identification of elevated SIRT3 levels in PBMCs in patients with advanced endometriosis who had undergone surgical intervention. The high inter-individual variability observed in SIRT3 protein levels in PBMCs, as reflected by the large standard deviations, is likely related to the limited sample size and the inherent biological heterogeneity of PBMCs. Therefore, it is crucial to acknowledge that further studies, with larger sample sizes and a control group, are imperative to corroborate and refine these findings.

### Strengths and Limitations

Strengths

1. Despite its limitations, to our knowledge, this is the first study comparing oxidative stress markers and regulators across different endometriosis phenotypes in infertile patients close and far from the oocyte, and in both cellular and liquid samples.
2. The study successfully identified an elevation of SIRT3 in PBMCs in patients with advanced endometriosis who had undergone surgical intervention.

Limitations

1. The absence of a control group limits the ability to make direct comparisons and draws attention to the need for caution in interpreting and generalizing the findings.
2. The small sample size is a significant limitation that may impact the statistical power and generalizability of the study results, emphasizing the importance of larger cohorts in future investigations.

## 4. Materials and Methods

We performed a longitudinal, prospective and unicentric cohort study. All the patients selected were diagnosed with endometriosis in the Endometriosis Unit at the Hospital Clínic of Barcelona (Barcelona, Spain). The inclusion criteria were infertile women less than 37 years of age, with OMA alone or a combination of DE + OMA (MIX), who underwent an IVF cycle in the Assisted Reproduction Unit of the Hospital Clínic of Barcelona and had a body mass index (BMI) between 18 and 25 and no consumption of toxic substances. Patients who did not meet the above requirements and patients with polycystic ovarian syndrome or any other chronic inflammatory disease were excluded.

Data related to the consumption of toxic substances such as tobacco, as well as food supplements or vitamin compounds (without medical prescription, except for the recommendation of folic acid) were recorded. Also, the occasional consumption of toxic substances was recorded in the last year.

Patients were asked whether they were receiving any hormonal treatment for endometriosis, and if so, what type of treatment they were undergoing. Specifically, they were inquired about the use of combined oral contraceptives, including whether they were taken in a cyclical or continuous regimen, progestogens, or the use of a levonorgestrel intrauterine device (IUD). For patients who were receiving hormonal treatment prior to the in vitro fertilization (IVF) cycle, the duration of the washout period before ovarian stimulation was recorded. By washout period, we refer to the interval between the discontinuation of the patient’s previous hormonal treatment and the beginning of ovarian stimulation for the in vitro fertilization process. Alternatively, it was noted whether they had initiated stimulation directly, without a break, by overlapping it with the ongoing IVF cycle.

It was also recorded whether the patients had undergone previous surgery for endometriosis.

All patients were offered study participation, and those willing to be enrolled signed the informed consent previously approved by the CEIm of our Hospital (code HCB/2019/1166). The study recruited patients for one year (September 2020–September 2021) and it finally included 20 patients with OMA, 20 patients with DE and 20 patients with the combination of OMA + DE.

**Figure 3:**
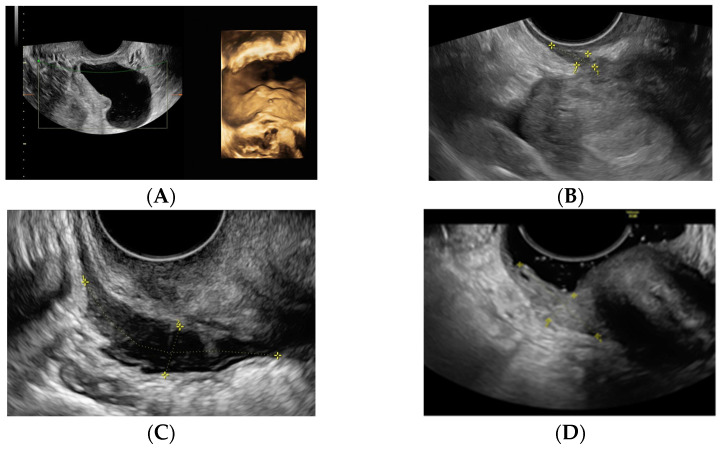

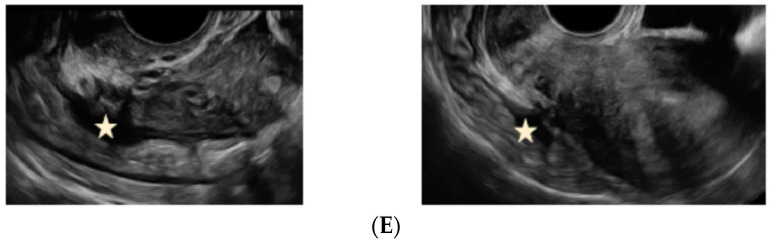
The first step was to recruit the endometriosis patients diagnosing them by expert sonographers with transvaginal ultrasound. 3D-transvaginal ultrasound images of different locations of deep endometriosis taken from our Endometriosis Unit at the Hospital Clínic of Barcelona ((**A**): bladder endometriosis nodule; (**B**): uterosacral ligament endometriosis nodule; (**C**): rectum wall nodule; (**D**): vagina wall nodule; (**E**): posterior compartment uterosacral ligament and rectum endometriosis nodule marked with an asterisk).

Diagnosis was made by transvaginal ultrasound (TVUS) performed by expert sonographers using an endovaginal probe (type RIC5-9, Voluson S10, General Electric, Seoul, Republic of Korea) and included 2-dimensional and 3-dimensional (3D) examinations. The presence of ovarian endometriosis and DE was assessed according to the International Deep Endometriosis Analysis (IDEA) group consensus [11]. The location of DE was described within the pelvis: rectosigmoid, rectovaginal septum, torus or uterosacral ligaments, vaginal fornix, or bladder (Figure 3).

OMA group was formed by patients with a sonographic diagnosis of either a single or multiple ovarian endometriomas, uni- or bilateral, without the presence of deep or peritoneal endometriosis. All diagnoses were made based on ultrasound performed by an experienced operator.

DIE group were patients with a sonographic diagnosis of deep endometriosis, defined as the presence of endometriosis lesions infiltrating the peritoneum >5 mm. This group is characterized by the presence of infiltrating nodules in the rectosigmoid, uterosacral ligaments, vaginal fornix, vaginal-rectal septum, and/or bladder [37].

MIX group were patients who presented both endometriomas and deep endometriosis lesions.

All patients underwent controlled ovarian stimulation for IVF procedure, and at the day of follicular punction, we also collected the samples for research. All the patients underwent the sterility study according on the protocols of the Assisted Reproduction Unit, including the determination of the ovarian reserve markers such as the antimullerian hormone (AMH) expressed in ng/mL, and antral follicular count (AFC). The IVF cycle was carried out according to usual practice, initiating a first phase of ovarian stimulation (with daily subcutaneous injection of 150 to 225 IU of follicle stimulating hormone according to the ovarian reserve and BMI) together with pituitary inhibition in a regimen of subcutaneous injection of 0.25 mg gonadotropin-releasing hormone antagonists daily initiated when the ovulation risk criteria were reached (estradiol > 400 pg/mL or follicular size > 14 mm). Triggering was done when the follicle reached >18 mm with 250 µgr of human chorionic gonadotropin (Ovitrelle → Merck Serono) or with 0.2 mg of triptorelin acetate subcutaneous (Decapeptyl → Ipsen). Follicular punction was performed under sedation and ultrasound guidance. Once the oocyte was obtained, it was decumulated and sperm microinjected for fertilization. Embryo transfer was performed after 3 or 5 days of embryo culture.

**Figure 4:**
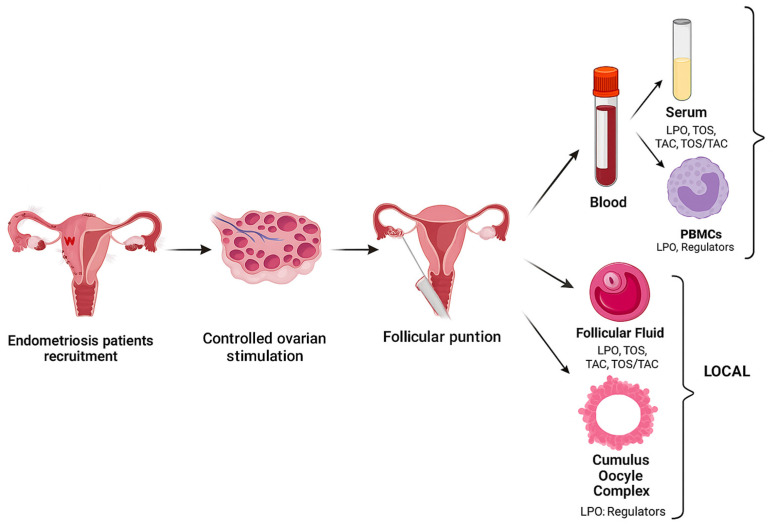
Steps of the methodology: first we recruited the endometriosis patients who met the inclusion criteria. Then we performed controlled ovarian stimulation and finally, on the day of the follicular punction, we collected the samples. We measured the different markers and regulators at a systemic and local level. LPO: lipid peroxidation; TOS: total oxidant status; TAC: total antioxidant capacity; PBMCs: peripheral blood mononuclear cells. Created with BioRender.com (accessed on 1 July 2024).

The samples were obtained the day of the follicular punction (blood, FF, and cumulus oocyte complex [COC]) (Figure 4).

### 4.1. Sample Preparation and Analyses (Figure 4)

Peripheral blood was collected by antecubital vein punction in 4 Vacutainer^®^ BD^®^ tubes: 2 tubes of 5 mL SST™ II Advance for serum collection, and 2 tubes of 10 mL EDTA K2 for plasma and peripheral blood mononuclear cell (PBMC) isolation. All tubes were centrifuged at 1500 for 15 min at room temperature. After this time, serum and plasma were separated from their respective tubes, and aliquots of 2 mL were extracted into cryotubes (Cry TubeTM Vials, Thermo Fisher Scientific, Madrid, Spain) for storage at −80 °C until the day of analysis.

For PBMC isolation, plasma-free blood was resuspended in 1× phosphate-buffered saline (PBS) solution (DPBS GIBCO, Thermo Fisher Scientific, Madrid, Spain) to reconstitute original volume, and added to a 50 mL FALCON tube (Fisher Scientific, Madrid, Spain) on the top of 10 mL of Ficoll (Lymphoprep, Fisher Scientific, Madrid, Spain). The mixture was then centrifuged for 30 min at 660 g to achieve separation of blood components based on their density. PBMCs were collected from the interface between plasma and Ficoll. After extraction using Pasteur pipettes, the interface was washed with PBS and centrifuged at 440 G for 10 min. The pellet obtained was resuspended in 2 mL of PBS and distributed into 4 aliquots, which were again centrifuged at 440 G for 10 min. After removing the supernatant, the PBMCs samples were stored at −80 °C until analysis.

Non-hemolytic FFs were collected after follicular punction and processed by centrifugation at 440 G at 4 °C, obtaining cell-free aliquots of granulosa that were frozen at −80 °C until analysis. To minimize oxidative stress during follicular fluid manipulation, the fluids were centrifuged at 440 g for 10 min at 4 °C. The combined effect of these three factors—low speed, short time and low temperature—minimizes the generation of oxidative stress during sample processing. Additionally, all liquids were processed in endotoxin-free polystyrene tubes (Vitrolife, Västra Frölunda, Sweden).

Regarding the COCs collected, they were washed in a protein-free medium (physiological serum) at 37 °C and distributed into 2 cryotubes (Cry TubeTM Vials, Thermo Nunc, Waltham, MA, USA), which were kept at −80 °C until analysis.

Cellular samples were sonicated to obtain a cell lysate, and total protein was quantified by bicinchoninic acid assay (BCA) following the manufacturer’s protocol (Thermo Scientific, #23227). Between two and four replicates per biological sample were included in all experimental assays except for Western blot quantification (based in one single measurement) due to limited sample availability.

Total Antioxidant Capacity: The total antioxidant capacity (TAC) is based on the reduction of copper (II) to copper (I) by antioxidants in the sample and measures intrinsic TAC power. The TAC assay was performed in 20 μL of patient serum and FF using an OxiSelect™ Total Antioxidant Capacity Assay kit (Cell Biolabs Inc., San Diego, CA, USA), and was quantified by spectrophotometry (maximum absorption at 490 nm). Results were expressed as μM copper reducing equivalents (CRE)/20 μL.

Total Oxidant Status: The total oxidant status (TOS) determines the overall oxidation state by quantifying the oxidation of ferrous ion into ferric ion by the oxidant molecules present in the sample. TOS was measured in 45 uL of patient serum and FF using the Total Oxidant Status Assay Kit (Kit-2173, Cerative Biomart, New York, NY, USA). The oxidative reaction was measured spectrophotometrically (absorption maximum at 530 nm), and results were expressed as μmol H^2^O^2^ equivalent per liter.

Lipid peroxidation in liquid samples: Lipid peroxidation (an indicator of oxidative damage produced by ROS to cellular lipid compounds) was quantified in 50 μL of serum and FF samples using the OxysResearch™LPO-586™kit (Deltaclon, Portland, OR, USA) through the spectrophotometric measurement of malondialdehyde (MDA) and 4-hidroxyalkenal (HAE), both of which are products of fatty acid peroxidation. Results were expressed as μM MDA + HAE/50 μL.

Lipid peroxidation in cellular samples: In case of PBMCs and COCs, the assay was also performed using the OxysResearch™LPO-586™kit (Deltaclon, Portland, OR, USA) in 20–40 ug of protein from the sample. Results were normalized by protein content and expressed as μM MDA + HAE/mg protein. 

Western blot (WB) for oxidative stress regulators: To assess the protein levels of oxidative stress regulators on cellular samples, the Western blot technique was performed. Total cell lysates (30 μg) from PBMCs and COCs were separated on 7/12% SDS-polyacrylamide gels and transferred at 20 V for 7 min using the iBlot2 Transfer system into a nitrocellulose membrane (iBlot 2 regular stacks; ThermoFisher IB23001, Madrid, Spain). These membranes were blocked with 5% skimmed milk during 1 h, and then immunoblotted overnight with the following primary antibodies (dilutions in 1% skimmed milk): 1:1000 SIRT3 (Cell Signaling #2627, Danvers, MA, USA), 1:500 FOXO3A (Cell Signaling #9467), 1:5000 β-actin (Abcam ab8227, Cambridge, UK), and 1:1000 SOD2 (ThermoFisher MA1-106). After incubation with the secondary antibodies: 1:2000 goat anti-mouse IgG-HRP (#170-6516 Biorad, Hercules, CA, USA), or 1:1000 anti-rabbit IgG HRP-linked (#7074 Cell signaling), specific bands were visualized by chemiluminescence (LAS400) and semi-quantified with ImageQuant TL analysis software v8.1. Results were normalized by β-actin to correct protein expression for sample loading.

### 4.2. Criteria for Deciding the Quality of Embryo

Between September 2020 and September 2021, embryos were preferentially transferred on the third day of culture.

Embryos were classified and selected for transfer or cryopreservation following the protocol of the Spanish Association for Studies in Reproductive Biology (ASEBIR), based on morphological characteristics (1, 2). Briefly, embryos were classified as type A (best quality, optimal implantation potential), B (good implantation potential), C (limited implantation potential) or D (worst quality, minimal implantation potential), according to the number of cells on day 2 and day 3, the percentage of fragments, multinucleation, presence/absence of vacuoles and the aspect of the zona pellucida [38].

### 4.3. Statistical Analysis 

Statistical analyses were performed using R software version 4.0.5 (R Core Team, 2020). Continuous variables are presented as means with standard deviations (SD) or as absolute values when appropriate, and categorical variables as frequencies and percentages. The Shapiro–Wilk test was applied to evaluate normality of continuous data distributions. For normally distributed variables, differences among study groups (OMA, DE, and MIX) were assessed using one-way analysis of variance (ANOVA). When multiple comparisons were necessary, *p*-values were adjusted with the Bonferroni correction to control for type I error. For non-normally distributed variables, the Kruskal–Wallis test was employed, as it does not assume normality and is appropriate for small sample sizes. Comparisons of categorical variables were conducted with the Chi-square (χ^2^) test, or with Fisher’s exact test when expected cell count were low. The significance level was set at *p* < 0.05 for all analyses.

Sample size was determined pragmatically according to the number of patients with endometriosis available in the assisted reproduction unit during the study period and based on sample sizes reported in related literature; therefore, no formal sample size calculation was carried out.

## 5. Conclusions

The conclusion of the study is that Sirtuin 3 (SIRT3) levels are significantly elevated in patients with more severe phenotype of endometriosis. These findings suggest a potential association between SIRT3 and the pathophysiology of endometriosis, with higher levels correlating with increased disease severity. Further research is needed to understand the role of SIRT3 in the progression of endometriosis and its potential as a biomarker for disease severity.

However, the absence of a control group and the small sample size emphasize the need for caution in generalizing our findings. Despite these limitations, this study highlights the complexity and heterogeneity in the literature regarding oxidative stress and endometriosis, reinforcing the importance of standardized methodologies and larger, diverse cohorts in future research.

## Figures and Tables

**Table 1:** Characteristics of the patients studied. Number of IVF cycles per patient, mean age and BMI. Ovarian reserve markers with AMH and AFC. Data are expressed as means ± standard deviation (SD). The differences between studied groups were determined with Kruskal–Wallis test (*p* < 0.05).

Variable	OMA	DE	MIX	*p*-Value (Kruskal–Wallis)
Number of cycles/patient	20	20	20	
Age (years)	34.58 ± 2.59	35.67 ± 2.23	36.40 ± 0.89	0.200
BMI (kg/m^2^)	22.73 ± 3.39	23.38 ± 2.33	20.81 ± 0.86	0.198
AMH (ng/mL)	3.49 ± 3.08	1.91 ± 1.23	1.33 ± 1.24	0.077
AFC	15.42 ± 7.59	9.25 ± 5.15	6.60 ± 3.71	0.010

**Table 2:** Pre-IVF treatments such as surgery, antioxidant intake and hormonal treatments. In case of previous hormonal treatment, the washout period was recorded. Continuous data are presented as mean ± standard deviation; categorical variables as *n* (%). Statistical comparisons among the three groups (OMA, DE, MIX) were performed using Fisher’s exact test. * Indicates statistical significance at *p* < 0.05. Significant differences were found in the proportion of patients with previous surgery (*p* < 0.001), previous hormonal treatments (*p* < 0.001), and washout periods (*p* < 0.001). Post hoc analysis revealed that the MIX group differed significantly from both OMA and DE groups in previous surgery and washout period distributions. Differences in previous antioxidant intake did not reach statistical significance (*p* = 0.128).

Variable	OMA	DE	MIX	*p*-Value
Previous surgery (%)	2 (10%)	11 (55%)	20 (100%)	<0.001 *
Previous antioxidant intake (%)	1 (5%)	3 (15%)	8 (40%)	0.128
Previous hormonal treatments (%)				<0.001 *
No previous treatment	14 (70%)	3 (15%)	8 (40%)	
Cyclic oral contraceptive pills	2 (10%)	9 (45%)	4 (20%)	
Progestogens	1 (5%)	3 (15%)	4 (20%)	
Continuous oral contraceptive pills	3 (15%)	5 (25%)	0	
Levonorgestrel-releasing IUD	0	0	4 (20%)	
Washout period (%)				<0.001 *
No previous contraceptive pills	14 (70%)	3 (15%)	8 (40%)	
Direct start	4 (20%)	13 (65%)	4 (20%)	
3 months	0	2 (10%)	0	
6 months	1 (5%)	0	0	
>12 months	1 (5%)	2 (10%)	8 (40%)	

**Table 3:** Outcomes of the IVF cycle with the final E2 value, the number of oocytes retrieved, the mature oocytes, the fecundated oocytes, the day which the embryos were transferred, the tax of useful embryos (transferrable embryos) and the pregnancy rate per transfer. Data are expressed as mean ± standard deviation for continuous variables and as percentages or proportions for categorical variables. Comparisons among groups (OMA, DE, MIX) were performed using the Kruskal–Wallis test for continuous data and Fisher’s exact test for categorical variables. * Indicates statistical significance at *p* < 0.05. A statistically significant difference was observed in the percentage of mature oocytes (MII) among groups (*p* = 0.039), with a lower proportion in the MIX group compared to OMA and DE. No statistically significant differences were found in final estradiol levels, number of oocytes retrieved, fertilization rates, embryo transfer characteristics, or pregnancy rates (all *p* > 0.05).

Variable	OMA	DE	MIX	*p*-Value
Final E2 (pg/mL)	2404.69 ± 1043.80	1994.82 ± 1250.71	1414.40 ± 697.34	0.176
No. oocytes retrieved	8.75 ± 4.01	7.18 ± 4.14	5.00 ± 2.83	0.194
% Mature oocytes (MII)	85.98	75.13	52.67	0.039 *
% Fertilized oocytes	51.98	56.42	57.50	0.717
Average no. embryos transferred	1.36 ± 0.77	1.55 ± 0.78	1.33 ± 0.58	0.655
Transfer day (%)				0.771
Day 3	16 (80%)	16 (80%)	20 (100%)	
Day 5	4 (20%)	4 (20%)	0	
% Useful embryo	66.43	73.94	55.29	0.836
Pregnancy rate per transfer	7/20 (35%)	6/20 (30%)	5/20 (25%)	0.787

**Table 4:** Oxido-reduction marker results of Lipid peroxidation, Total Antioxidant Capacity and Total Oxidant Status in the different samples. Data are presented as mean ± standard deviation. Comparisons among groups (OMA, DE, MIX) were performed using the Kruskal–Wallis test for non-parametric continuous variables. No statistically significant differences were observed among the groups for any of the oxidative stress biomarkers measured in follicular fluid (FF), serum (SRM), PBMCs, or cumulus–oocyte complexes (COCs), including lipid peroxidation (MDA + HAE), total oxidant status (TOS), and total antioxidant capacity (TAC) (all *p* > 0.05).

Variable	OMA	DE	MIX	*p*-Value
Lipid peroxidation (µM MDA + HAE)				
Mean FF	0.1186 ± 0.05	0.1182 ± 0.05	0.1187 ± 0.06	0.951
Mean Serum	0.13 ± 0.04	0.13 ± 0.07	0.10 ± 0.06	0.384
Mean PBMCs	3.40 ± 1.27	3.63 ± 2.05	3.05 ± 1.50	0.911
Mean COCs	9.29 ± 7.15	7.91 ± 7.53	17.09 ± 13.43	0.439
Total oxidant status (µmol H_2_O_2_ Equiv./L)				
Mean FF	4.28 ± 4.28	4.24 ± 0.79	3.38 ± 0.48	0.103
Mean SRM	4.78 ± 1.78	5.38 ± 3.17	3.83 ± 0.36	0.074
Total Antioxidant Capacity (µM CRE)				
Mean FF	60.11 ± 12.53	62.18 ± 12.43	55.92 ± 20.31	0.927
Mean SRM	76.62 ± 12.62	85.31 ± 32.72	67.93 ± 4.32	0.321

**Table 5:** Logistic regression analysis to assess the parameters that could be related to the elevation of SIRT3. * Indicates statistical significance at *p* < 0.05.

Variable	OR (Odds Ratio)	CI (Confidence Interval)	*p*-Value
Antioxidants	2.023	22.477–18.430	0.839
Pre-IVF hormonal treatment	2.880	5.5507–11.268	0.483
Previous surgery	8.919	0.569–17.269	0.037 *

## Data Availability

The original contributions presented in this study are included in the article/Supplementary Material. Further inquiries can be directed to the corresponding author.

## Supplementary Materials

The following supporting information can be downloaded at: https://www.mdpi.com/article/10.3390/ijms26189110/s1.
