# Supplementary material for: Exploring Oxidative Stress in Different Endometriosis Phenoptypes: Insights from Ovarian and Systemic Perspectives by the Study of SIRT3
Source: Int J Mol Sci. 2025 Sep 18;26(18):9110. doi: 10.3390/ijms26189110 (PMC12470309; doi:10.3390/ijms26189110)

Supplementary files

	OMA	DE	MIX	P-value
SIRT3 COCs	7.042±7.69	12.013±15	10.419±7.67	0.373
SOD2 COCs	3.587±3.255	4.063±4.65	8.111 ±11.975	0.985
FOXO3A COCs	3.324±6.214	1.749±1.253	2.37±1.80	0.61
SIRT3 PBMCs (WB)	1.528±0.638	2.055±0.785	3.210 ±2.229	0.038
SOD2 PBMCs (WB)	3.314±0.892	5.285±3.656	4.975 ±2.411	0.363
FOXO3A PBMCs (WB)	0.141±0.126	0.156±0.103	0.284 ±0.275	0.338

Table S1. Oxidative stress regulators results of SIRT3, SOD2 and FOXO3A in COCs and PBMCs. Data are presented as mean ± standard deviation. Comparisons among groups (OMA, DE, MIX) were performed using the Kruskal–Wallis test due to non-normal distribution of the data. A statistically significant difference was observed in SIRT3 protein levels in PBMCs ($p = 0.038$), with the MIX group showing higher mean expression compared to OMA and DE. No significant differences were found among groups in the expression levels of SOD2 or FOXO3A in PBMCs (all $p > 0.05$).

Figure S1.

PBMCs western blots:

Blot A:

Lane number	Sample
1	OMA
2	Failed sample
3	OMA
4	Failed sample
5	CTL
6	Protein standards (molecular weight)
7	CTL
8	CTL
9	CTL
10	DIE
11	DIE
12	DIE
13	MIX
14	Positive control
15	Positive control

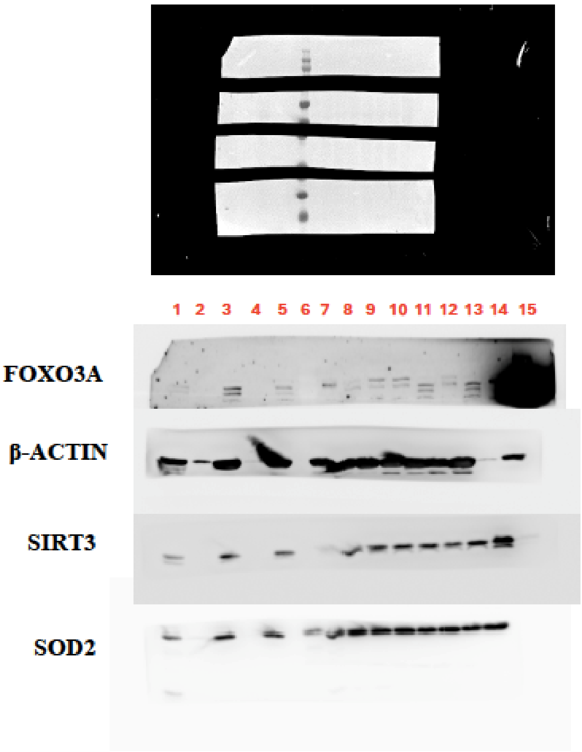

Blot B:

Lane number	Sample
1	Positive control
2	Positive control
3	Protein standards (molecular weight)
4	OMA
5	OMA
6	OMA
7	OMA
8	CTL
9	CTL
10	CTL
11	CTL
12	DIE
13	DIE
14	DIE
15	MIX

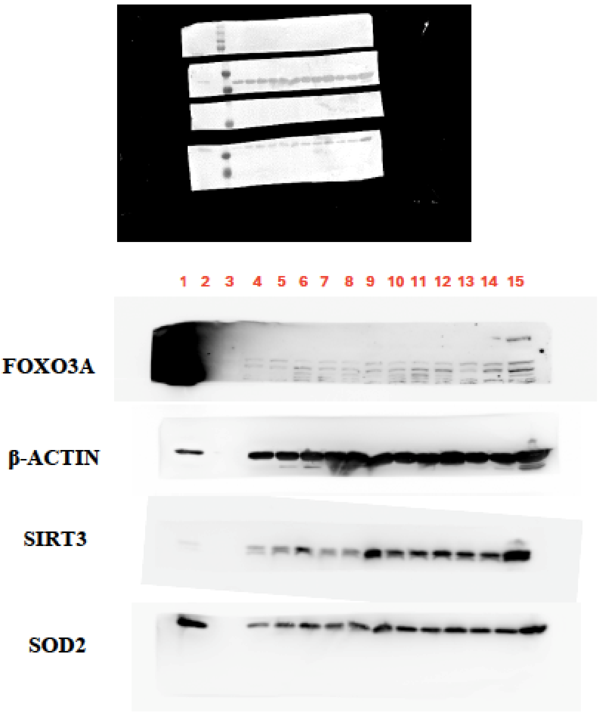

Blot C:

Lane number	Sample
1	Positive control
2	Protein standards (molecular weight)
3	OMA
4	OMA
5	OMA
6	OMA
7	CTL
8	CTL
9	CTL
10	CTL
11	DIE
12	DIE
13	DIE
14	MIX
15	Loading control

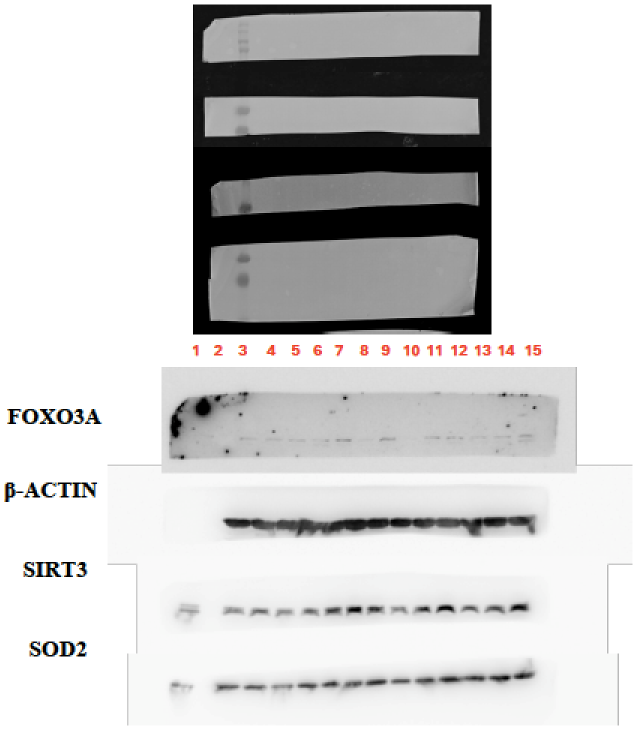

Blot D:

Lane number	Sample
1	Positive control
2	OMA
3	OMA
4	Protein standards (molecular weight)
5	OMA
6	OMA
7	CTL
8	CTL
9	OMA
10	OMA
11	DIE
12	DIE
13	MIX
14	MIX
15	Loading control

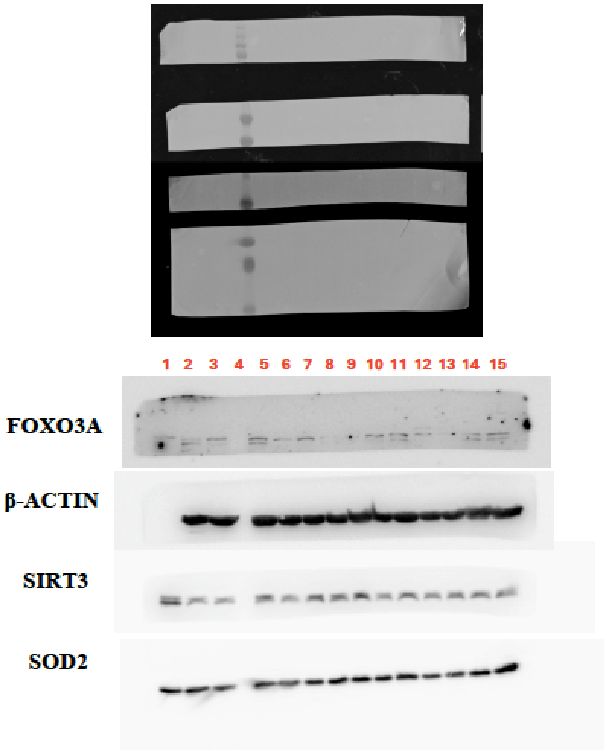

COCs western blots:

Blot E:

Lane number	Sample
1	Positive control
2	Protein standards (molecular weight)
3	OMA
4	OMA
5	OMA
6	CTL
7	CTL
8	CTL
9	CTL
10	Failed sample
11	DIE
12	MIX
13	Loading control

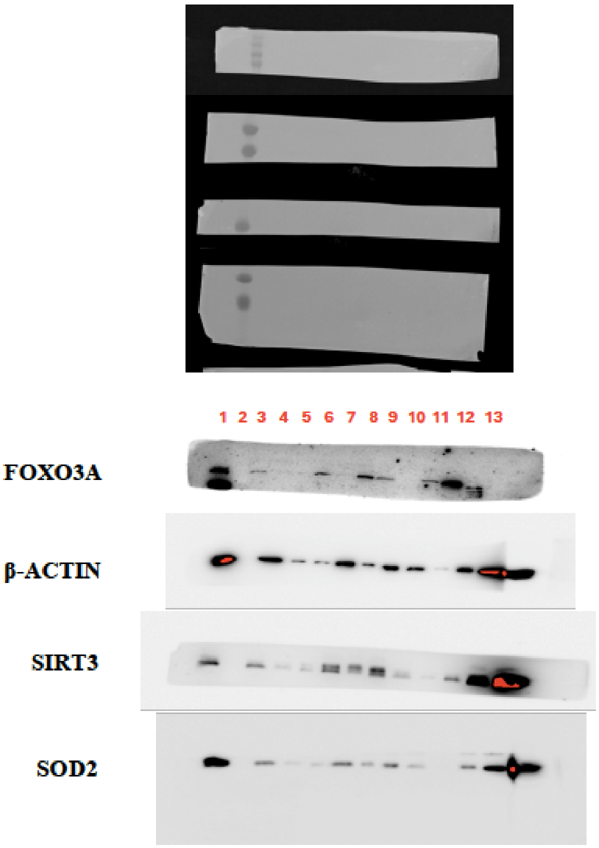

Blot F:

Lane number	Sample
1	Positive control
2	OMA
3	OMA
4	OMA
5	OMA
6	CTL
7	Protein standards (molecular weight)
8	CTL
9	CTL
10	CTL
11	DIE
12	DIE
13	DIE
14	MIX
15	Loading control

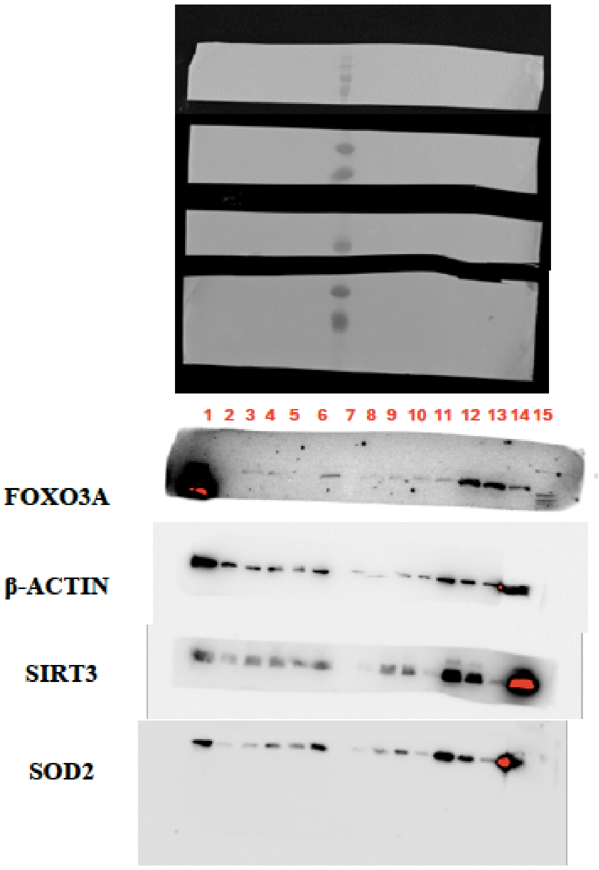

Blot G:

Lane number	Sample
1	Positive control
2	Protein standards (molecular weight)
3	OMA
4	OMA
5	OMA
6	OMA
7	OMA
8	OMA
9	CTL
10	CTL
11	CTL
12	CTL
13	DIE
14	DIE
15	Loading control

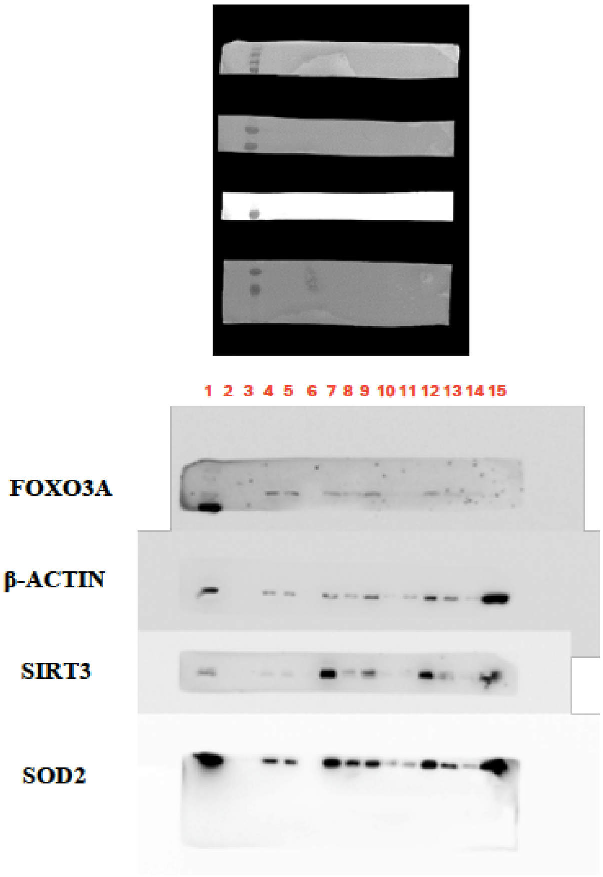

Blot H:

Lane number	Sample
1	Positive control
2	OMA
3	OMA
4	OMA
5	OMA
6	OMA
7	Protein standards (molecular weight)
8	CTL
9	CTL
10	CTL
11	OMA
12	DIE
13	MIX
14	MIX
15	Loading control

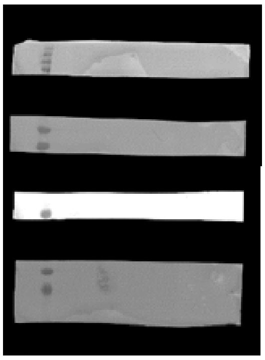

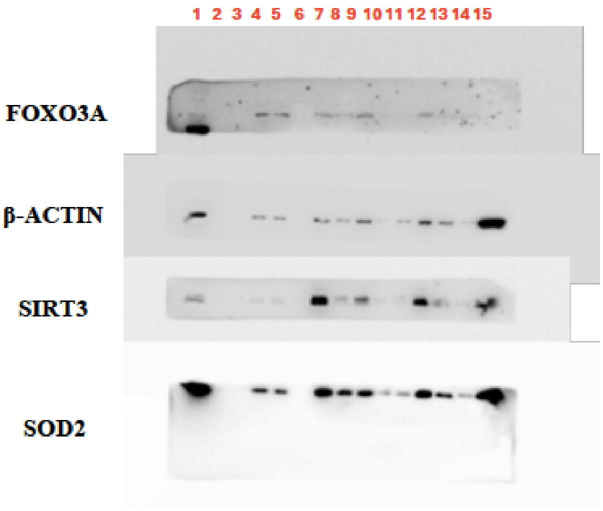

Supplement: Supplementary file 1 [file ijms-26-09110-s001.zip › ijms-3816556-supplementary.pdf]
